# Instrumentation for Routine Analysis of Acrylamide in French Fries: Assessing Limitations for Adoption

**DOI:** 10.3390/foods10092038

**Published:** 2021-08-30

**Authors:** Mark M. Skinner, Jared T. Seale, Maranda S. Cantrell, Joseph M. Collins, Matthew W. Turner, Owen M. McDougal

**Affiliations:** 1 Department of Chemistry and Biochemistry, Boise State University, Boise, ID 83725, USA; markskinner@u.boisestate.edu (M.M.S.); jaredseale@u.boisestate.edu (J.T.S.); 2 Biomolecular Sciences Graduate Programs, Boise State University, Boise, ID 83725, USA; marandacantrell@u.boisestate.edu (M.S.C.); josephcollins177@u.boisestate.edu (J.M.C.); 3 Biomolecular Research Center, Boise State University, Boise, ID 83725, USA; matthewturner1@boisestate.edu

**Keywords:** acrylamide, quantitation, liquid chromatography–mass spectrometry, gas chromatography–mass spectrometry, near-infrared spectroscopy

## Abstract

The purpose of this experimental review was to detect acrylamide in French fries using methods most adaptable to the food process industry for quality control assessment of products. French fries were prepared at different cook times using the same fryer oil over a five-day period to assess the influence of oil degradation and monitor trends in acrylamide formation. Acrylamide detection was performed using LC-MS, GC-MS and FT-NIR. The low levels of acrylamide produced during frying, low molecular weight of the analyte, and complexity of the potato matrix make routine acrylamide measurement challenging in a well-outfitted analytical lab with trained personnel. The findings of this study are presented from the perspective of pros and cons of each acrylamide measurement method in enough detail for food processors to appraise the method that may work best for them based on their available instrumentation and extent of personnel training.

## 1. Introduction

**Scheme 1:**
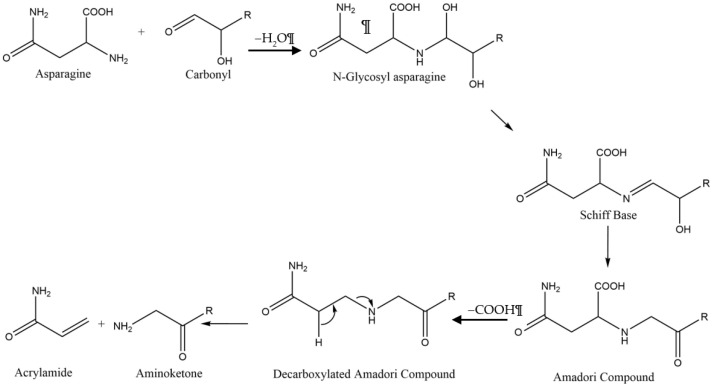
The combination of asparagine and a reducing sugar at elevated temperatures produces acrylamide via the Maillard reaction.

In rodent studies, high levels of acrylamide have proven to be neurotoxic, potentially carcinogenic, and cause impairment of the male reproductive system [1,2,3,4]. In 2002, acrylamide was discovered in a variety of fried foods and subsequently, the state of California instituted regulations that mandate acrylamide warning labels for food and beverages, while the European union has implemented acrylamide suppression strategies in food production to limit acrylamide content to below 750 µg/kg in food and beverages [5,6,7,8]. According to the California Office of Environmental Health Hazard Assessment (OEHHA), acrylamide is listed as causing cancer and reproductive toxicity. The California OEHHA lists a “no significant risk level” of cancer at 0.2 µg/day and a “maximum allowable dose level” for reproductive toxicity of 140 µg/day. As of 2017, the European Union has instituted mitigation strategies for all food business operators which produce foodstuffs known to contain acrylamide [5]. The EU has instituted acceptable benchmark levels of acrylamide which vary depending on the foodstuff in production with the benchmark for French fries set at 500 μg of acrylamide per kg of “ready to eat” French fries [7]. Foods and beverages like French fries, potato chips, roasted coffee beans, crackers and many others are produced by frying or baking at elevated temperatures (Table 1), which provide conditions to convert asparagine and reducing sugars into acrylamide via the Maillard reaction (see Scheme 1) [8,9]. French fries and potato chips can easily exceed the threshold acrylamide levels set by the EU, and measurement strategies are required to comply with government regulation [10].

The state of Idaho is the largest grower and processor of potatoes and potato products in the United States of America (USA), responsible for 29% of all potato products sold annually [11]. The Snake River basin is one of the most fertile soil regions in the USA, with optimal climate conditions for potato production. Raw tubers prior to cooking are acrylamide free, but contain asparagine and reducing sugars that generate acrylamide upon heating to temperatures above 120 °C [8]. A major market for potato and potato product sales is California, the fifth largest economy in the world, with a gross domestic product of USD 2.75 trillion [12]. The addition of acrylamide warning labels on food and beverages in California has inspired a movement within the food processing industry, both in Idaho and across the country, to minimize and monitor acrylamide levels in processed food products.

Recent reviews describe emerging methods for acrylamide measurement, but with the exception of FT-NIR, many of these techniques are not feasible in an industrial setting due to the complexity of the methods and need for well-trained personnel [10,13,14]. LC- and GC-MS are the most commonly employed methods of acrylamide quantitation, but recently FT-NIR has been proposed as a viable quality assurance method [15]. FT-NIR is commonly used for food analysis and many industry laboratories are equipped with FT-NIR spectrometers. FT-NIR methods developed for acrylamide quantification would be well-suited for potato processing facilities and thus provided motivation for the present study. French fries were selected for analysis because acrylamide levels tend to be elevated for this product [10]. Additionally, correlation of acrylamide concentration to fryer oil quality has been included in the current investigation.

LC-MS studies are the most commonly adopted method for acrylamide analysis in food. Gökmen et al. investigated the relationship between acrylamide formation and cooking time and temperature in French fries using LC-APCI-MS. Results from this study indicated that acrylamide formation was most prevalent on the surface of the fry with concentrations of 72, 2747, and 6476 μg/kg when French fries were cooked for 9 min at 150, 170, 190 °C, respectively [16]. In general, the sample preparation for acrylamide analysis by LC-MS begins with a cooked food that is freeze dried, ground to a powder, and extracted with water and/or acetonitrile. For fried products such as French fries, defatting is commonly implemented using a nonpolar solvent like hexanes. The filtered extract is analyzed by LC-MS in accordance with well-established methods [10,17]. For effective quantitation, an internal standard like acrylamide-d3 or acrylamide (1,2,3-^13^C_3_) should be used when performing LC- or GC- methods. The LC-MS instrument of preference used by commercial chemical analysis laboratories for acrylamide quantitation is a triple quadrupole mass spectrometer (TQMS), which is often not available in food process laboratories [17,18,19]. The experiments conducted for the present study were performed using Q-TOF single quadrupole MS instrument and detail the benefits and challenges associated with this more common, less expensive instrument.

Acrylamide is a poor analyte for study by GC-MS due to its low molecular weight and short retention time through solid support columns. To further complicate GC-MS methods, acrylamide is known to polymerize at temperatures above 138 °C and decompose between 175 and 300 °C [20,21]. Sample preparation for GC-MS analysis of acrylamide proceeds according to a similar protocol as LC-MS, but derivatization by bromination to produce 2,3-dibromopropionamide (2,3-DBPA) is conducted prior to analysis to suppress polymerization and increase detection of the higher *m*/*z* derivatized analyte [8,10]. The instrument of choice for commercial lab quantitative analysis of acrylamide in food samples is GC-MS with a triple quadrupole mass spectrometer (TQMS) [22]. Using GC-TQMS, acrylamide can be directly measured without derivatization under ideal conditions at concentrations in the range of 1–1000 ng/mL [22], or from potato matrix extraction with bromination at a limit of detection of 6.94 µg/kg and a limit of quantitation of 20.83 µg/kg [23]. In the absence of a GC-TQMS, the results presented here reflect analysis obtained on a less expensive, more commonly available, single quadrupole GC-MS.

In contrast to the well-established LC- and GC-MS acrylamide measurement options, there is substantial interest and advantage to the creation of a reliable FT-NIR method [15]. FT-NIR is widely used in the food industry because it is rapid, easy to use, and economical relative to LC or GC-MS. Furthermore, personnel training and sample preparation for FT-NIR instrumentation is far less involved than LC- or GC-MS. In the food processing industry one distinct advantage to NIR spectroscopy over LC- or GC-MS is that potato flour samples can be analyzed directly, without the need for acrylamide extraction or derivatization.

The three methods for detection reviewed in this study include liquid chromatography–mass spectrometry (LC-MS), gas chromatography–mass spectrometry (GC-MS) and Fourier transform–near infrared spectroscopy (FT-NIR). The intent of the present study was to identify the pros and cons to conducting routine acrylamide analysis using instrumentation that can be feasibly adopted in an industrial laboratory setting. In order to evaluate LC-MS, GC-MS and FT-NIR methods for the determination of acrylamide in French fries, it was necessary to prepare samples for analysis in such a way as to address fundamental questions on the topic of acrylamide formation. This study used commercially available frozen par-fried French fries, prepared by frying in unused fryer oil obtained from a local limited liability corporation (LLC) restaurant. To address how cook time affects acrylamide formation, separate French fry samples were cooked according to the product label as well as overcooked for extended periods of time This study was intended to address how the extended cook time affects acrylamide formation, and determine the ability to extract acrylamide from the potato matrix for quantitative analysis. Potato frying trials were repeated each day for five days to evaluate the influence of oxidative degradation of fryer oil on acrylamide formation. As vegetable-based fryer oils degrade, the extent of oxidative deterioration can be assessed by measurement of oil polymerization by high pressure liquid chromatography (HPLC), free fatty acid content by titration, oxidation product formation using UV-Vis spectroscopy, and measurement of total polar materials gauged with a Testo [24,25,26]. To incorporate a distinct endpoint for fryer oil lifespan, a sample of oil was obtained from the local restaurant at time of disposal. This heavily used oil was black in comparison to the straw yellow color of unused oil. The waste oil was used to fry batches of French fries at times of 4, 8 and 16 min, where the recommended cook time according to the manufacturer was 4 min. This later study attempted to follow the acrylamide measurement in a time-course experiment to correlate cook time to acrylamide formation and assess the influence of oil quality on acrylamide content.

## 2. Materials and Methods

### 2.1. Acrylamide Standards

Acrylamide monomer (0.02 g, 0.28 mmol, TCI Chemicals, 98.0%) was added to acetonitrile (200 mL, Fisher Scientific, HPLC grade) to make a stock solution. Serial dilutions to the stock solution were prepared at concentrations of 0–10,000 µg/L in 500 µg/L increments. 2,3-DBPA standard (Alfa Aesar^TM^, Haverhill, MA, USA, 97%) was used for GC-MS calibration. Acrylamide-d3 (500 mg/L in acetonitrile, analytical standard grade, Fisher Scientific, Burlington, VT, USA) was used as an internal standard for LC-MS analysis.

### 2.2. French Fry Sample Preparation

Standard cut grade A French fries made from US grown potatoes were purchased from the freezer section of a local grocery store and stored at −20 °C. In the first trial 15 portions of 715.0 g of fries were weighed for each of the corresponding days and time periods described below, followed by submersion in a Presto ProFry^TM^ (Model 0546616, USA) deep fryer containing unused vegetable oil at 175 °C, and the fries were cooked for the product label recommended time of 4 min. The fries were removed from the oil and a new batch of fries were introduced to cook for 8 min, and the third batch of fries were cooked for 16 min. The following day, another three batches of fries, were cooked for 4, 8 and 16 min in the same oil as the prior day. This process was repeated for a total of five consecutive days. The Presto ProFry^TM^ was set to a temperature of 175 °C for all trials. The fryer automatically adjusts the temperature using a variable resistor such that the temperature remains constant throughout the experiment. Fryer oil levels were monitored throughout the duration of the experiment to ensure sufficient levels for fries to be submerged or free floating at all times. No oil addition was required throughout the duration of the study. An additional batch of fries was cooked in waste oil donated by the same local restaurant that provided the original unused fryer oil. Three sets of French fries were cooked in the waste oil for 4, 8, and 16 min. Oil was not added or replaced after each frying session over the five-day period, however, ample oil was present throughout the course of the study. Following each batch of fries being removed from the deep fryer, the samples were cooled, diced, and soaked in liquid nitrogen, dehydrated in a FreeZone 2.5 (LABCONCO, Kansas City, MO, USA) lyophilizer for 16 h, and finely ground into potato flour using a Mr. Coffee, Coffee Mill PN: 18373 (Sunbeam Products INC., Boca Raton, FL, USA) coffee grinder. A second trial was performed using a batch size of 100.0 g of French fries. The preparation of French fries was the same as those used for the first trial, with the only procedural changes being cook times for eight batches of fries at 2, 4, 6, 8, 10, 12, 14 and 16 min and frying taking place over a single day. The Presto ProFry^TM^ fryer, unused oil, and consistent volume of oil that were used at the start of the first study were replicated for the second trial.

### 2.3. GC-MS Sample Preparation

#### 2.3.1. Bromination of Acrylamide in French Fry Flour

French fry flour (5.0 g) from each sample batch of the initially lyophilized 715 g (i.e., 4, 8 and 16 min on days one through five) was placed into a clear colorless volumetric flask and filled to the 100.0 mL mark with deionized (DI) water. The flour was solubilized by sonication for 5 min followed by stirring for 30 min. The solution was filtered through a Whatman grade GF/A filter paper using a Buchner funnel (11.0 cm diameter). A 25.0 mL portion of the filtrate was transferred to a separatory funnel, into which was added 25.0 mL of n-hexane (Acros Organics, Carlsbad, CA, USA, 99+%) to extract residual oil from the sample. The separatory funnel was stoppered, inverted to mix contents, and vented repeatedly over a period of two minutes. Bromination of the extracted acrylamide was conducted according to the method of Surma et al., 2017 [27]. Specifically, the aqueous bottom layer was drained into a 125 mL actinic Erlenmeyer flask, into which was added potassium bromide (Fisher Scientific, USA, 99%) (7.5 g, 6.3 × 10^−2^ mol), followed by saturated bromine water (saturated at 20 °C reagent grade, Fisher Scientific, USA) (8.0 mL) with stirring. The bromination reaction mixture was put into an ice bath and stored at 0 °C for 1.5 h in the dark. The solution was transferred to a 125 mL separatory funnel, and 10–15 drops of aqueous 1.0 M sodium thiosulphate solution (J.T. Baker, Phillipsburg, NJ, USA, >99.0%) was added to neutralize the remaining bromine, indicated by obtaining a clear colorless solution. Ethyl acetate (Fisher Scientific, USA, 99.9%) (25.0 mL) was then added to the separatory funnel and the mixture homogenized by inversion over a time period of one minute. The organic phase (top layer) was isolated and dried with 1.0 g of anhydrous sodium sulphate (Acros Organics, USA, 99%) prior to filtration through a 0.45 µm nylon membrane filter (Thermo Scientific, Waltham, MA, USA) into an auto sample GC-MS vial with a polytetrafluoroethylene (PTFE) lid.

#### 2.3.2. GC-MS Instrument Method

Sample data were acquired using a Thermo Scientific^TM^ ISQ^TM^ 7000 GC-MS system programed according to the method detailed by Surma et al., 2017 [28]. In brief, the carrier gas was helium and the injection port temperature was set to 260 °C. A 1.0 µL injection volume was volatilized for sample loading onto the TG5-SilMS column (Thermo Scientific cat. #26096-1420, 0.25 mm I.D., 0.25 µm film thickness, 30 m length), which was held at 60 °C for one min, then ramped 20 °C/min to 200 °C, held for 3 min at 200 °C, then ramped 30 °C/min to 250 °C, and held for 5 min at 250 °C. The GC-MS sample injector and detector interface temperatures were held at 260 °C. A calibration curve was generated for 2,3-DBPA (Alfa Aesar^TM^, USA, 97%) over a concentration range of 1 µg/kg to 1000 mg/kg to assess the limit of detection and limit of quantitation of the instrument. An extracted ion analysis protocol was applied using the Chromeleon^TM^ 7.2 software with a determined LOD of 1090 μg/kg and LOQ of 3310 μg/kg.

### 2.4. LC-MS Sample Preparation

LC-MS sample preparation involved the use of a QuEChERS extraction method. A 1.0 g sample of the initially lyophilized 715 g (i.e., 4, 8 and 16 min on days one through five) freeze dried French fry potato flour was added to a centrifuge tube (50 mL), followed by addition of 5.0 mL of hexanes (Fisher, USA, 99.9%) to extract fryer oil from the sample. DI water (10.0 mL) and acetonitrile (10.0 mL) were then added to the tube, forming a triphasic solution. The addition of 4.0 g of magnesium sulphate (Fisher, Japan, anhydrous certified powder) and 0.5 g sodium chloride (Fisher, USA, 100.0%) increased the polarity of the aqueous layer for a more efficient extraction of residual oil (hexane) and to provide a prominent interface for the biphasic solution interface between water and acetonitrile. Inversion of the mixture with intermittent pressure release over 1 min was followed by 5 min of centrifugation at 5000 rpm. A 1.0 mL aliquot of the acetonitrile layer was transferred into a 1.5 mL microcentrifuge tube followed by addition of 0.150 g magnesium sulphate and 0.05 g of PSA solid phase extraction (SPE) bulk sorbent (Agilent Technologies, Santa Clara, CA, USA, HPLC grade). The solution was vortexed for 30 s to ensure thorough mixing and residual particulate was separated by centrifugation at 5000 rpm for 1 min. The supernatant was collected and passed through a 0.45 µm nylon membrane filter into an HPLC vial for analysis. French fry flour (50 g) from the second set of French fry samples was placed into an Erlenmeyer flask with hexanes (100 mL), deionized water (50 mL), and acetonitrile (50 mL). The French fry mixture was sonicated for 30 min, then vigorously stirred for an additional 30 min. After stirring, the mixture was filtered via vacuum filtration and the filtrate was transferred to a separatory funnel. Magnesium sulfate (4 g) and sodium chloride (2.5 g) were added to the triphasic filtrate, followed by shaking for 1 min, venting occasionally to relieve pressure. The aqueous layer (bottom) and acetonitrile layer (middle) were separated into two 50 mL centrifuge tubes and centrifuged (5000 rpm, 5 min). The acetonitrile layers were combined and concentrated to ~5 mL via rotary evaporation. The concentrated solutions were then dried in 1.5 mL Eppendorf tubes by SpeedVac (Thermo Fisher, Waltham, MA, USA) to remove remaining solvent. Dried samples were re-dissolved in water (250 µL). A 90 µL aliquot of the re-dissolved solution was spiked with 10 µL of acrylamide-d_3_ standard.

#### 2.4.1. LC-MS Instrument Method

LC-MS was performed with an Ultimate 3000 uHPLC system (Thermo Fisher Scientific, Waltham, MA, USA) equipped with either an Allure Acrylamide analytical column (2.1 × 50 mm, 5 µm) (Restek Corporation, Bellefonte, PA, USA) or an XTerra^®^ MS C_18_ column (2.1 × 150 mm, 3.5 µm) (Waters Corp., Milford, MA, USA) preceding an ultra-high resolution Quadrupole Time of Flight (Q-TOF) instrument (Bruker maXis, Bruker Corporation, Billerica, MA, USA). LC provided separation of analyte with a column temperature set to 30 °C. The mobile phase A was 0.1% formic acid in 18 MΩ nanopure water and phase B was 0.1% formic acid in acetonitrile. The injection volume was 5 µL and the flow rate 0.2 mL/min. For the Allure Acrylamide column, a linear gradient method was used starting at 100% phase A and ending at 60% phase B over 2.5 min. Phase B was maintained at 60% for 2 min and then increased to 80% for an additional 3 min. For the XTerra^®^ column, Phase B was initially set to 5%, and then increased to 90% over a 40 min linear gradient. A standard curve was created using a commercially available acrylamide (Tokyo Chemical Industry, Portland, OR, USA, 98.0%) standard from 20 to 2000 mg/kg, each spiked with 100 mg/kg acrylamide-d_3_ (500 mg/L in acetonitrile, analytical standard grade, MilliporeSigma Supelco, Bellefonte, PA, USA) through either the Allure or XTerra^®^ columns, to determine which column provided the lowest limit of detection and limit of quantitation.

The mass spectrometer was operated under the following conditions: electrospray ionization (ESI) using positive ion mode; nebulizer pressure: 1.2 Bar; flow rate of drying gas (N_2_): 8 L/min; drying gas temperature: 200 °C; voltage between HV capillary and HV end-plate offset: 3000 V to −500 V; mass range was set from 50 to 500 *m*/*z*; the quadrupole ion energy was 4.0 eV. Sodium formate was used to calibrate the system in the mass range. Data were analyzed using the the Compass Data Analysis software v. 4 (Bruker Corporation, Billerica, MA, USA).

### 2.5. NIR Sample Preparation

NIR samples were prepared by adding potato flour (1.0 g), of the initially lyophilized 715 g (i.e., 4, 8 and 16 min on days one through five), to proprietary scintillation vials (Bruker, Billerica, MA, USA). A Bruker MPA FT-NIR was used in integrating sphere mode to take and overlay spectra of the 18 dry French-fried potato flour samples with the number of scans set to 32. Partial Least Squares (PLS) software was used to compare GC-MS data to acrylamide concentrations taken with FT-NIR in a linear regression model. A multi-variant calibration algorithm, (PLS), was applied to the data using the Bruker OPUS software to correlate spectral intensity in the 5000–5500 and 6000–6500 cm^−1^ regions of the NIR spectra to concentration values for the acrylamide standards. The calibration for experimental samples was obtained from GC-MS results.

### 2.6. Fryer Oil Analysis

#### 2.6.1. Polymerization of Oil

Normal phase size exclusion high performance liquid chromatography was used to determine polymerization of triglycerides following IUPAC standard method 2.508. Approximately 0.05 g of each fryer oil sample was passed through a 0.45 µm nylon filter into a 1.5 mL HPLC sample vial. To the filtered oil was added 1.0 mL of tetrahydrofuran (HPLC grade, Fisher, USA). An isocratic normal phase HPLC method was used with tetrahydrofuran as the mobile phase and a flow rate of 1.0 mL/min. The stationary phase was a GPC/SEC column PL1110-6525 (Agilent Technologies, USA) preceding a Dionex Corona Veo RS charged aerosol detector (Thermo Scientific, USA). All samples were run in triplicate.

#### 2.6.2. Free Fatty Acid Determination

Free fatty acids were analyzed using the AOCS official Method Ca 5a-40. A class A 100 mL burette was used in the titration of oil samples for each of the five days of the study, and for the heavily used oil from the local restaurant. Approximately 3.0 g of oil was pipetted into a 250 mL Erlenmeyer flask along with hot ethanol (75 mL, 95%) and phenolphthalein indicator (2 mL, 1% in 95% ethanol). The mixture was vigorously shaken until an emulsion formed. A sodium hydroxide solution (0.001 M) was prepared and standardized using 6 M hydrochloric acid. The sodium hydroxide solution was poured into the burette and the emulsion was titrated until a faint pink color remained for at least 30 s. The acid value was calculated in accordance with the AOCS method [25].

#### 2.6.3. p-Anisidine Value Measurement

p-Anisidine value was analyzed in each fryer oil sample as an indication of oxidative degradation of the oil. Aliquots of the fryer oil from each of the five days of the study and the heavily used restaurant oil were treated with sodium sulphate to remove residual water. The sodium sulphate was eliminated by filtration through a 0.45 µm nylon filter. A standard solution was prepared by adding 0.5 g of p-anisidine (Tokyo Chemical Industry, Japan, (98.0%) to a 200.0 mL volumetric flask that was then filled to volume with glacial acetic acid (Alfa Aesar, Mixcoac, Mexico, 99+%). Filtered fryer oil (0.5 g) was transferred into a 25.0 mL volumetric flask and filled to volume with isooctane (Fisher Chemical, USA, 99.0%). The oil solution (5.0 mL) was pipetted into one test tube and combined with 1.0 mL of p-anisidine standard solution. The solution was allowed to react for 10 min. After exactly 10 min, an absorbance reading of the solution was acquired at 350 nm using a Cary 60 UV-Vis spectrophotometer (Agilent Technologies, USA). The p-anisidine value was determined in triplicate for each time point, for each sample batch, at each day, and again with the heavily used oil sample from the local restaurant.

#### 2.6.4. Total Polar Material Determination

Total polar materials (TMP) from fryer oil taken each day for five days, and again with heavily used oil from a local restaurant, were measured using a Testo 270 deep-frying oil tester (Testo, Inc., Sparta, NJ, USA).

## 3. Results

### 3.1. GC-MS

A calibration curve was created using a standard solution of 2,3-DBPA over the concentration range of 0.1–100 mg/kg, which gave a linear correlation with an R^2^ of 0.9989. Acrylamide extracted from French fries, and derivatized to 2,3-DBPA, was measured by GC-MS to be between 4.96 and 14.81 mg/kg of French fry flour (Table 2). An acrylamide level detected by GC-MS of 14.81 mg/kg is higher than literature values but may be due to the fries being cooked four times longer than the supplier’s recommended time. The French fries cooked for 16 min were notably overdone, darkly colored, and extra crispy. It was observed that as the frying time of the French fries increased from 4 to 8 to 16 min, the concentration of acrylamide generally doubled. An effect on acrylamide formation from continuous use of the same frying oil was not observed throughout the five-day frying period. Additionally, the concentration of acrylamide in French fries cooked in the highly degraded local restaurant oil did not vary greatly from the values for French fries cooked in the lab. The limit of detection (LOD) and limit of quantification (LOQ) for 2,3-DBPA analysis by the GC-single quadrupole mass spectrometer were determined to be 1.09 and 3.31 mg/kg, respectively. All experimental acrylamide measurements were above these limits. T-tests were performed on GC-MS values for day 1, 15 min and day 5, 8 min and these values did not pass with a *p*-value less than 0.05 so they were not reported as results for these experiments (i.e., results noted as ND in Table 2).

### 3.2. LC-MS

A calibration curve using acrylamide-d_3_ was formed over the range of 50–2000 mg/kg with an R^2^ of 0.9988. The LOD and LOQ for the LC-MS method using the Allure Acrylamide analytical column was 27 and 83 mg/kg, respectively. The French fry derived potato flour samples were extracted using the quick, easy, cheap, effective, rugged, and safe (QuEChERS) extraction method with an additional concentration step on a SpeedVac to improve acrylamide detection and increase the signal-to-noise ratio on the Q-TOF. Acrylamide concentration in the first set of French fries was between 3.64 and 9.75 mg/kg (Table 3) and increased with frying time up to 8 min, however, this trend did not continue for samples cooked for 16 min. Unlike the values determined using GC-MS, the concentration of acrylamide at 16 min decreased to levels below those observed at shorter frying times. This result may be due to caramelization of the overcooked French fries. Increased caramelization at longer cooking times is expected to have resulted in less efficient extraction, translating to lower observed acrylamide concentrations in the final samples.

The results in Table 2 and Table 3 provide a five-day average acrylamide detection by GC-MS for samples cooked for four minutes to be 6.0 mg/kg, whereas the same five-day measurement by LC-MS provided an average acrylamide level of 6.0 mg/kg. When cook times were extended to eight minutes, the average acrylamide levels detected using the GC-MS protocol over the same five-day period was 8.0 mg/kg, and the LC-MS method measured the acrylamide levels at an average of 7.6 mg/kg. Since no observed influence of oil degradation on acrylamide formation was noted, a subsequent experiment was conducted where fries were cooked from 2 to 16 min, at batch sizes of 100 g, and batch cook times were conducted every two minutes for eight batches. Lower than expected values at 16 min fry times is attributed to inefficient acrylamide extraction from overcooked and caramelized French fries [29,30]. The results of this later experiment were assessed by LC-MS.

For the second LC-MS trial, a Waters^®^ XTerra column was used in place of the Allure Acrylamide analytical column, because it was discovered that lower LOD and LOQ could be obtained. A calibration curve using acrylamide-d_3_ was generated over the range of 50–2000 mg/kg with an R^2^ of 0.9927. The LOD and LOQ using the Waters^®^ XTerra column was 14 and 40 mg/kg, respectively, roughly half the levels as the Allure Acrylamide column. The ratio of peak areas between acrylamide and acrylamide-d_3_ at each frying time for the second set of French fry samples (100 g, trial 2) were assessed from 2 to 16 min in 2 min increments. Acrylamide was detected in French fries that were analyzed directly from the bag (t = 0 min), which demonstrates low levels of acrylamide production during par frying of the consumer potato products. Unfortunately, this detection value was qualitative and not quantitative since the quantities of acrylamide in uncooked fries was below the LOD of 14 mg/kg. The acrylamide level in the French fries increased from the two-minute fry time, and an upward trend in acrylamide concentration with respect to frying time was observed up to 8 min. After 8 min, the acrylamide levels became unreliable to measure and by 14 min, the extraction process was no longer effective.

### 3.3. NIR

**Figure 1:**
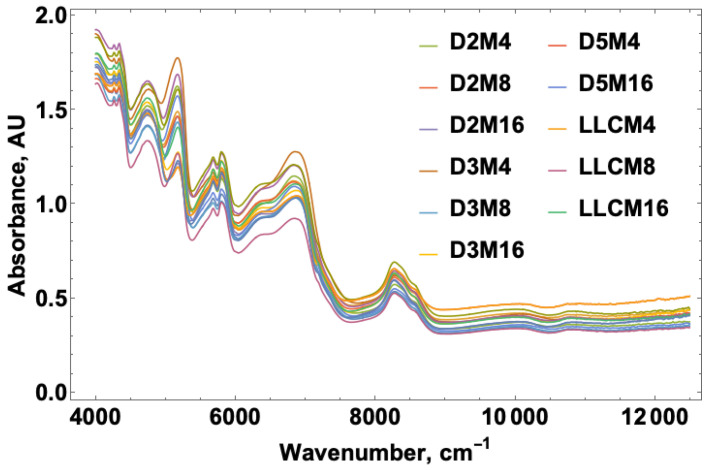
NIR spectral overlay from 4000 to 12,000 cm^−1^ for acrylamide levels over a five-day study and fry times at 4, 8 and 16 min on each day. D = day, M = minute, LLC = LLC oil provided from a local restaurant.

NIR spectra were acquired for the French fry samples obtained at 4, 8, and 16 min fry times over the course of five days and included a sample set using the heavily used fryer oil from the local restaurant. Figure 1 shows the spectral overlay for the NIR data over the range from 4000 to 12,000 cm^−1^. The figure legend provides labels where D corresponds to the day, M refers to the cook time in minutes, and LLC represents the heavily used restaurant fryer oil. The portion of the spectrum studied for acrylamide corresponded to the 5000–5500 and 6000–6500 cm^−1^ regions representing the carbonyl (C=O) and primary amide (N-H) overtones, respectively.

**Figure 2:**
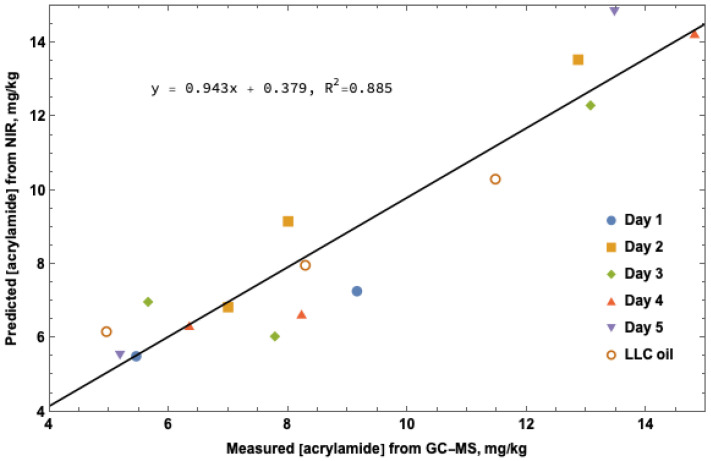
NIR acrylamide prediction. Predicted acrylamide concentration from NIR spectral data as compared to actual acrylamide concentration in mg/kg measured by GC-MS.

NIR spectral analysis using the partial least squares algorithm was conducted with the GC-MS acrylamide values to validate quantitation for NIR prediction. Figure 2 shows the correlation between GC-MS measured acrylamide levels and software predicted acrylamide levels from spectral interpretation. A correlation between increasing cook time and acrylamide concentration is apparent, however a significantly larger sample set will be required to obtain useful data for reliable NIR measurement.

### 3.4. Fryer Oil

#### 3.4.1. Polymerization of Triglycerides

The polymerization of triglycerides was measured by HPLC over the five days of the fryer trial and compared to the heavily used oil obtained from the LLC. Figure S1 shows the appearance of polymerized triglycerides that increase in concentration with cook time. Interestingly, the heavily used LLC oil shows similar levels of triglyceride polymer as day five fryer oil used in this study.

#### 3.4.2. Free Fatty Acid

The concentration of free fatty acids, represented by acid value, was found to increase within the fryer oil for each day of oil use, as would be expected for fryer oil compromised by thermal degradation. Although the acid value increased for each day, the heavily used LLC oil showed a notably higher level of free fatty acids than the oils used in the lab trial. This result is supported by the extent to which the restaurant oil was used for frying significantly greater product quantities within the 4–5 day period before disposal. The results of the free fatty acid titration study are provided in Table 4.

#### 3.4.3. p-Anisidine Value

UV-Vis results for p-anisidine value in fryer oil over the course of the study showed a similar systematic increase with oil degradation. The highest p-anisidine value was obtained from the heavily used LLC oil (see Table 4).

#### 3.4.4. Total Polar Materials

Total polar material measurement by hand-held Testo approximated percent levels that increased with each subsequent day of cooking from 8.0% on day one up to 12.5% by day five. The TPM content in the LLC oil was found to be slightly lower than the day five result at 11.5%, which may be due to addition of fresh oil over time as oil levels drop due to product absorbance in the restaurant setting. The TPM percent measurements are provided in Table 4.

## 4. Discussion

### 4.1. GC-MS

The results from the single quadrupole GC-MS using an extracted ion analysis method were conclusive that oil degradation had no effect upon the formation of acrylamide, but an increase in cook time at a temperature of 175 °C with the same oil directly correlated to an increase in acrylamide content. The extracted ion analysis permitted identification of mass to charge ratio of ++207.85 *m*/*z* (base peak) for 2,3-DBPA. Since the mass spectrometry settings were positive ion mode, the M+ peak would have been +231.89. The selected extracted ion model used isolated ions based upon the mass to charge ratio of the base peak and was able to detect the presence of acrylamide in experimental samples in the range of 4.96–14.81 mg/kg. In every case, longer cook times of 8 and 16 min resulted in higher acrylamide content in French fries from an average of 5.92 mg/kg at 4 min to 8.92 mg/kg at 8 min, and less reliable results at 16 min (see Table 2).

Besaratinia et al. reported mean acrylamide concentration in French fries as 334 µg/kg with maximum concentration of 5.312 mg/kg [31]. In the present study, French fries analyzed using GC-MS contained acrylamide at concentrations averaging 5.92 mg/kg for fries cooked at the recommended temperature of 175 °C and four-minute time. Cook times were extended to 8 and 16 minutes to track the influence of time on acrylamide concentration, and a correlation was observed where longer times led to more acrylamide formation.

The data in this study appears at times to be nearly one order of magnitude higher than the data in Gökmen et al. Although all temperatures and cook times in this paper were stringently adhered to, a hallmark of the Gökmen paper was that an increase in temperature rapidly affects acrylamide production. Since the cook time in this paper is 5 °C higher than the mid-range temperature of the Gökmen paper, only a qualitative comparison is reasonable in this instance. Furthermore, different products were used in this study. It was determined during the second LC-MS quantitation that the uncooked fries had an existing acrylamide concentration due to a par frying method used in the production of the product. This consideration as well as other variables could explain why the results in this study yielded a higher amount of acrylamide when compared to the results of the Gökmen et al. paper. To assess the influence of oil degradation on acrylamide formation, fryer oil was monitored over time for triglyceride polymer content, free fatty acid formation, p-anisidine value, and percent total polar materials. The result of this analysis did not provide data supporting the effects of oil degradation on acrylamide formation, in agreement with previous studies [32,33]. There was a notable increase in every measure of oil degradation component, but acrylamide levels were consistent between limited use oil and heavily used oil.

In this study, acrylamide from French fries was quantified to a LOD and LOQ of 1090 and 3310 μg/kg, respectively. Given the observed values for acrylamide were found to be in excess of 4.96 mg/kg, the GC single quadrupole MS was sufficient for the analysis. Literature reports of GC-electron capture detector (ECD), GC-TQMS, or GC-flame ionization detector (FID) rather than a single quadrupole mass spectrometer provide precedent for decreasing the LOD and LOQ for acrylamide extracted from fried potato products. EPA method 8032A utilizes GC analysis of 2,3-DBPA by GC-ECD. Previous studies using GC-ECD to quantify 2,3-DBPA in fried potato products reported LOD and LOQ of 0.1 μg/kg and 3 μg/kg, values below those of our experimentally measured amounts [34,35]. When ECD is used in combination with GC, additional precautions must be taken regarding column type and method of derivatization. ECD is a halogen sensitive detector, therefore, derivatization of acrylamide to 2-bromoprop-2-enamide (2-BPA) or 2,3-DBPA aids in achieving LOD and LOQ lower than those observed by LC-MS/MS [34]. Zhang et al. noted that the HP-INNOWax column provided the greatest separation of acrylamide from co-extracted species in comparison to weakly polar or non-polar GC columns such as DB-5 and DB-23 [35]. Studies using GC-TQMS to quantify 2-BPA from brominated French fry extracts obtained LOD and LOQ of 6.94 and 20.83 μg/kg, respectively [23]. GC-TQMS has shown potential for quantifying acrylamide in water (1–1000 ng/L) without derivatization [22]. Eliminating the derivatization steps would greatly reduce the time required to analyze samples. Literature regarding GC-FID as a method for quantifying acrylamide is sparse, however, these studies present potential for optimizing the technique. Over a seven-day period, Pedersen et al. performed a Soxhlet extraction with methanol to obtain acrylamide before GC-FID analysis [36]. Acrylamide concentration in potato chips was reported at 14,500 μg/kg with a standard error of 6%, but LOD and LOQ were not reported [36]. Due to the time requirement for this method, Ghiasvand et al. sought to optimize acrylamide extraction with headspace solid-phase microextraction for the purpose of GC-FID analysis [37]. Percent recoveries ranged from 79.6 to 95.7% with LOD and LOQ of 220 and 770 μg/kg, respectively. For both GC-FID studies, acrylamide derivatization was not required. A comparison of GC-FID to GC-TQMS and GC-ECD shows that the LOD and LOQ for a GC-FID is one to two orders of magnitude higher, however, eliminating derivatization saves time and introduces fewer steps that may compromise quantitation.

Acrylamide bromination to generate 2,3-DBPA was a rigorous method used for GC-MS that proved to be more effective than QuEChERS for LC-MS, especially for overcooked fries. The GC-MS protocol called for extended extraction of the potato material through three to four hours of bromination, extraction and sample matrix penetration of overcooked, caramelized and oil saturated French fry flour. The extended time for bromination and solvent exposure provided extraction conditions leading to a more linear correlation between cook time and acrylamide formation as compared to the LC-MS extraction method. More effective extraction from the longer cooked fries at 16 min is evident in the greater concentrations of acrylamide with a mean value of 13.5 ± 0.9 mg/kg. This is significantly greater than the 4 min mean value of 6.0 ± 0.8 mg/kg and 8 min mean value of 8.0 ± 0.2 mg/kg. The primary shortcomings of the GC-MS method used in this study stems from the inability to attain limits of detection and quantification below 1 mg/kg. Three to four hours are required to prepare, extract, brominate, and run each sample on the GC-MS. In comparison to other methods described in this study, GC-MS proved to be the most accurate method but proved to be the slowest method for acrylamide analysis due to the bromination protocol.

### 4.2. LC-MS

Two HPLC columns were evaluated to determine the LOD and LOQ for each. For the Restek Allure Acrylamide column, the LOD and LOQ were determined to be 27 and 83 mg/kg, respectively, while the Waters^®^ Xterra column provided LOD and LOQ values of 14 and 40 mg/kg, respectively. Although there is a marginal difference in the LOD and LOQ observed for each, no definitive conclusion can be reached about whether either column outperformed the other. This is because different LC methods were used for each column (see Section 2.4.1). It is most likely that the slight increase in sensitivity observed on the Waters^®^ Xterra column is due to the extended LC gradient that helped minimize the influence of matrix effects such as ion suppression. The QuEChERS method does not have the intensity or have a heating requirement to exceed the glass transition temperature (T_g_) to break up the polysaccharides and release all the trapped acrylamide, which may have led to poor extraction efficiency results attained by the LC-MS methods. This is evident when comparing the LC-MS data to the GC-MS data. A calculated mean value of the five-day trial at 4 min for both LC-MS and GC-MS were both roughly around 6.0 mg/kg. The mean concentration value for the samples at 8 min was 8.0 mg/km for GC-MS and 7.6 mg/kg for the LC-MS. The most obvious instance of lesser extraction efficiency occurring in this experiment is at 16 min where the GC-MS mean concentration is 13.5 mg/kg and LC-MS is 4.7 mg/kg.

### 4.3. NIR

Using 16 dry potato flour samples taken on integrating sphere platform and calculated using OPUS/QUANT PLS software, the results from the spectral overlays analyzed at 5000–5500 and 6000–6500 cm^−1^ conform to the linear model of predicted vs. true acrylamide concentration. These 16 samples proved the concept that PLS software could be used to quantitate acrylamide in potato flour obtained from fried potato product. Absorbance readings measured at 5000–5500 and 6000–6500 cm^−1^ corresponded to the roto-vibrational energy of the first overtones of the sp^3^ hybridized N-H and carbonyl (C=O) stretch for the amide functional group in acrylamide, respectively. To further improve the PLS software (OPUS/QUANT, Billerica, MA, USA) analysis, four times the number of samples would need to be collected. The 18 points on the predicted over true linear regression model (Figure 2) provided an excellent proof of concept for acrylamide concentration prediction using the PLS model and optimizing processes of vector normalization (SNV) and first derivative. To implement the use of FT-NIR measurement of acrylamide in an industrial setting, acquisition of spectra for at least 80–100 samples coupled with wet sample quantitation of acrylamide through LC-MS or GC-MS would be required.

The GC-MS data (Table 2) was used to establish the values for the proof-of-concept curve (Figure 2) above. Data points from the 4 min cook times over days one, two, four and five; 8 min LLC; 16 min LLC, days three and four were right on the calibration curve. The remainder of the points were close enough to the calibration curve to show a trend and therefore validate the proof of concept. All three of the methods of GC-MS, LC-MS and FT-NIR used in this experiment yielded proof that the values near the four min mark were consistent and accurate. The GC-MS and LC-MS data were consistent at 8 min except for the 8 min LLC oil with an LC-MS concentration of 4.56 mg/kg and GC-MS 8.29 mg/kg. The difference in these two values trends in favor of the concept of varying extraction efficiency of the GC-MS bromination versus the LC-MS QuEChERS method. The FT-NIR concentration for the 8 min LLC sample is remarkably close to the GC-MS value of 8.29 mg/kg.

### 4.4. Fryer Oil

#### 4.4.1. Triglyceride Polymerization via SEC Normal Phase HPLC

Fryer oil was analyzed for polymerization of triglycerides by HPLC-CAD using an isocratic normal phase method and a size exclusion column. Increases in the concentration of polymers were shown by measuring a dirty oil peak in both LLC and experimental oil (6.6 min) 3.8 times larger than the new oil peak at 6.6 min (Figure S1). The observation that triglyceride polymers were more abundant in fryer oil that was heavily used was expected. The question we attempted to address was whether the high polymer content oil accelerated acrylamide formation in French fries. The result of increased triglyceride polymerization showed no effect on acrylamide production in French fried potatoes. The lack of data supporting the effects of oil degradation on acrylamide formation is in agreement with previous studies [36,37].

#### 4.4.2. Free Fatty Acid Content via Titration

The concentration of free fatty acids increased in the analyzed fryer oil as it degraded with use, over the five-day period of this study. Titrations were performed for oil obtained on each day, which reflected a gradual increase in acid value. The fresh vegetable oil started with an acid value of 0.275 mg NaOH/g of oil prior to use and increased over the five days where frying took place. The acid value of the oil after the fifth day of frying was 0.621 mg NaOH/g of oil thus verifying the breakdown of triglycerides. The increase in free fatty acid content in the oil as it degraded showed no correlation to an increase in the amount of acrylamide produced in the French fried potatoes.

#### 4.4.3. p-Anisidine Values via UV-Vis

Oxidation levels in oil are an indication that a given oil used for frying may be experiencing a loss in quality. One way to test oxidation levels in fryer oil, specifically the formation of unsaturated aldehydes, is to react p-anisidine with the oil in iso-octane to form light yellow oxidation products. The concentrations of the oxidation products were determined by measuring UV-Vis absorbance values (λ = 350 nm). From the UV-Vis study, it was observed that the absorbance representing oxidation products tripled over the course of the five-day study period starting at 4.43 AU/g on day zero and ending at 50.43 AU/g. The heavily used oil from the local LLC had spectra with the highest absorbance values at λ = 350 nm, corresponding to nearly a four-fold increase in oxidation products at 62.02 AU/g as compared to day one values of 17.12 AU/g after one cook time (see Table 4). The increase in oxidation levels indicates that oil becomes degraded with extensive use. However, according to our previous results, oil degradation exhibited little to no effect on acrylamide formation in French fries.

#### 4.4.4. Total Polar Materials via Testo 270

As the oil samples for each day were analyzed, the percent of total polar material incrementally increased from 8.0 percent on day one to 11.5 percent on day five. The acrylamide content of the French fries did not correlate despite increasing TPM percent in the oil.

## 5. Conclusions

The preferred instrumentation for acrylamide analysis is triple quadrupole mass spectrometers coupled to either a gas chromatograph or liquid chromatograph. The sample preparation for acrylamide extraction has been well studied and follows a QuEChERS protocol or variant of it. GC-MS studies routinely rely on bromination of acrylamide to 2,3-DBPA to obtain sufficient sensitivity to measure the low levels of acrylamide present in food products. FT-NIR spectroscopy has many advantages to GC-MS or LC-MS methods with respect to instrument expense, sample preparation, data acquisition, and result processing. However, NIR spectroscopy requires a robust dataset and validation by GC-MS or LC-MS. While potentially promising, NIR utility remains underdeveloped for acrylamide analysis to date. The current study demonstrated the limitations of a GC-single quadruple mass spectrometer, which included a substantial derivatization step to brominate acrylamide, and a LOD of 1.09 mg/kg, when food analysis needs to quantitatively measure acrylamide in the 50–500 µg/kg range. Analysis of acrylamide using an LC-Q-TOF MS instrument provided LOD of 14 mg/kg, which although derivatization was not required, the quantitation level was not sufficiently sensitive for routine analysis. The use of GC-MS to measure acrylamide concentrations for the present study provided results that were used to establish a partial least squares predictive analysis of NIR data for acrylamide in French fry potato flour. NIR results demonstrate the desired linear correlation, consistent with GC-MS measurement, indicative of the potential for NIR to gain widespread usage for quality assurance and quality control evaluation of food products. Lastly, the exploration of oxidative degradation of fryer oil showed increases in polymers, p-anisidine value, free fatty acids and total polar materials, however, the degraded oil did not influence acrylamide production in French fries. One interesting result was a higher value for acrylamide content in par-fried potatoes as they are known to be blanched during processing, which lowers the amount of reducing sugars, a precursor to acrylamide formation. Future directions for this study include determining sugar levels before frying and correlating these values to acrylamide formation.

## Figures and Tables

**Table 1:** Average levels of acrylamide found in food products according to 2015 data adapted from the FDA: https://www.fda.gov/food/chemical-contaminants-food/survey-data-acrylamide-food (accessed 29 August 2021).

Food Product	Average Acrylamide (μg/kg)
French fries	337
Potato chips	739
Coffee beans (ground)	272
Crackers	234

**Table 2:** GC-MS values for acrylamide concentration (mg/kg) per frying time for French fries. ND: not determined.

Usage	Acrylamide Concentration (mg/kg) per Frying Time		
	4 min	8 min	16 min
Day 1	5.45	9.16	ND
Day 2	6.99	8.00	12.86
Day 3	5.65	7.78	13.07
Day 4	6.34	8.23	14.81
Day 5	5.18	ND	13.41
LLC oil	4.96	8.29	11.48

**Table 3:** LC-MS values for acrylamide concentration (mg/kg) per frying time from French fries.

Usage	Acrylamide Concentration (mg/kg) per Frying Time		
	4 min	8 min	16 min
Day 1	7.33	9.75	5.69
Day 2	6.69	7.37	5.21
Day 3	7.22	6.49	3.64
Day 4	4.61	6.70	5.23
Day 5	7.23	7.56	3.77
LLC oil	4.65	4.56	4.98

**Table 4:** Values obtained in fryer oil for acid value, p-anisidine and total polar materials.

Usage	Acid Value (mg NaOH/g of Sample)	p-Anisidine Value (AU/g)	Total Polar Materials (%)
Day 0	0.275 ± 0.009	4.43 ± 0.03	--
Day 1	0.301 ± 0.011	17.12 ± 0.02	8.0
Day 2	0.309 ± 0.005	26.72 ± 0.04	8.5
Day 3	0.353 ± 0.005	38.88 ± 0.03	8.5
Day 4	0.482 ± 0.033	45.15 ± 0.02	11.0
Day 5	0.621 ± 0.017	50.43 ± 0.05	12.5
LLC oil	0.986 ± 0.044	62.02 ± 0.01	11.5

## Data Availability

Data contained in the manuscript.

## Supplementary Materials

The following are available online at https://www.mdpi.com/article/10.3390/foods10092038/s1, Figure S1. Oil polymerization of triglycerides over a 5-day period. After 5 days, the oil is typically replaced with fresh oil for frying. The arrow indicates the increased presence over time of a new peak at a retention time of approximately 6.45 min at a rate of 0.71 area units per day, indicative of a decrease in oil quality.
