# Supplementary material for: Instrumentation for Routine Analysis of Acrylamide in French Fries: Assessing Limitations for Adoption
Source: Foods. 2021 Aug 30;10(9):2038. doi: 10.3390/foods10092038 (PMC8469642; doi:10.3390/foods10092038)
Supplement: Supplementary file 1 [file foods-10-02038-s001.zip › supplementary material.pdf]

Supplemental Materials

Figure S1 shows the appearance of polymerized triglycerides that increase in concentration with cook time. Interestingly, the heavily used LLC oil shows similar levels of triglyceride polymer as day 5 fryer oil used in this study.

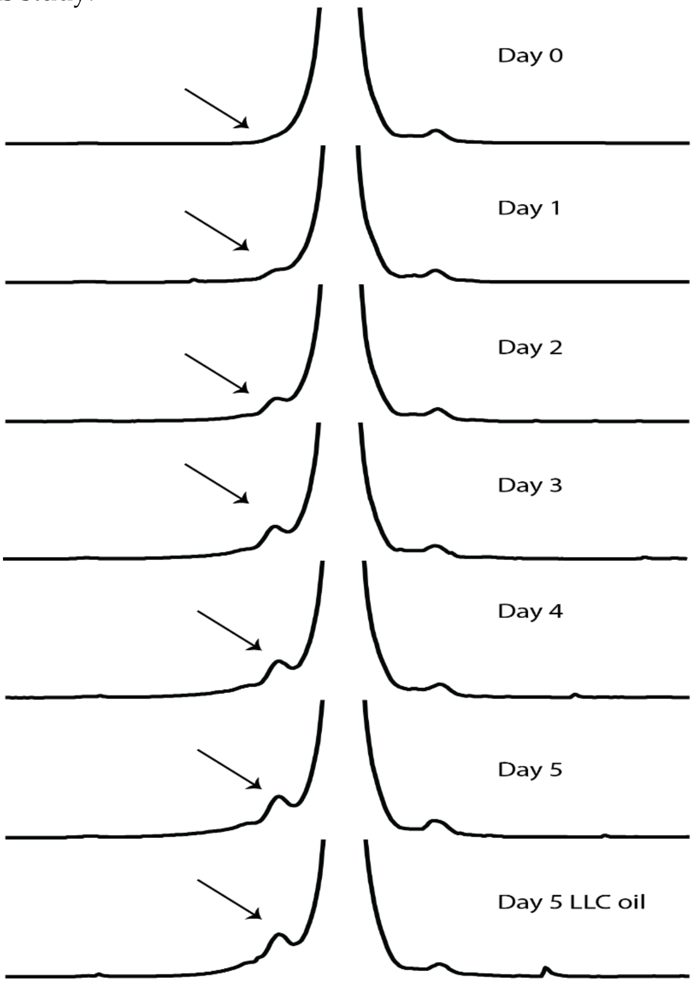

Figure S1. Oil polymerization of triglycerides over a 5-day period. After 5 days, the oil is typically replaced with fresh oil for frying. The arrow indicates the increased presence over time of a new peak at a retention time of approximately 6.45 min at a rate of 0.71 area units per day, indicative of a decrease in oil quality.
